# Cre Fused with RVG Peptide Mediates Targeted Genome Editing in Mouse Brain Cells In Vivo

**DOI:** 10.3390/ijms17122104

**Published:** 2016-12-14

**Authors:** Zhiyuan Zou, Zhaolin Sun, Pan Li, Tao Feng, Sen Wu

**Affiliations:** 1 State Key Laboratory for Agrobiotechnology, College of Biological Sciences, China Agricultural University, Beijing 100193, China; zzythinker@126.com (Z.Z.); szl305@126.com (Z.S.); lipan19900504@163.com (P.L.); taocau@163.com (T.F.); 2 College of Life Science, Liaoning University, Shenyang 110036, China

**Keywords:** genome editing, RVG peptide, Cre, blood brain barrier

## Abstract

Cell penetrating peptides (CPPs) are short peptides that can pass through cell membranes. CPPs can facilitate the cellular entry of proteins, macromolecules, nanoparticles and drugs. RVG peptide (RVG hereinafter) is a 29-amino-acid CPP derived from a rabies virus glycoprotein that can cross the blood-brain barrier (BBB) and enter brain cells. However, whether RVG can be used for genome editing in the brain has not been reported. In this work, we combined RVG with Cre recombinase for bacterial expression. The purified RVG-Cre protein cut plasmids in vitro and traversed cell membranes in cultured Neuro2a cells. By tail vein-injecting RVG-Cre into Cre reporter mouse lines mTmG and Rosa26^lacZ^, we demonstrated that RVG-Cre could target brain cells and achieve targeted somatic genome editing in adult mice. This direct delivery of the gene-editing enzyme protein into mouse brains with RVG is much safer than plasmid- or viral-based methods, holding promise for further applications in the treatment of various brain diseases.

## 1. Introduction

Cell penetrating peptides (CPPs) are typically five to 30 amino acids long, with the ability to penetrate mammalian cell membranes. By covalent binding, non-covalent binding or fusion expression, CPPs can facilitate cellular the entry of macromolecules such as proteins, nanoparticles, and drugs [1,2,3,4]. The need for more efficient means to deliver biological macromolecules in vivo has generated a broad interest in CPPs. An increasing number of CPPs have been identified in screens using phage display or mRNA display [5,6]. RVG, a CPP from a rabies virus glycoprotein, is particularly interesting because it can cross the blood-brain barrier (BBB) and enter brain cells, possibly through binding the nicotinic acetylcholine receptor without compromising the BBB [7,8].

The RVG peptide has been successfully used to carry a variety of cargos into brain cells such as plasmids [2], siRNAs [7], proteins [9], and nanoparticles [10]. However, targeted delivery of genome-editing Cre recombinase with RVG into the brain has not been reported. Cre protein is a 38 kD site-specific recombinase found in phage P1, and it has become a powerful tool for gene editing in the mouse genome [11,12]. In the current study, we first examined the targeting ability of RVG-Cre in cultured neuronal cells. We next systemically delivered RVG-Cre via tail vein injection into two Cre reporter mouse lines to examine its in vivo brain targeting ability, and demonstrated that RVG-Cre can target the brain and mediate efficient genome editing in somatic cells.

## 2. Results

### 2.1. RVG-Cre Properties In Vitro

**Figure 1:**
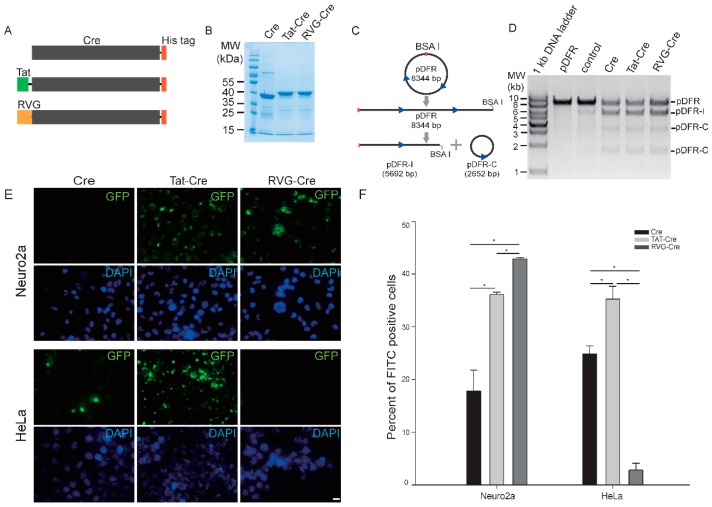
Purity, activity, and cell specificity of RVG-Cre, Tat-Cre, and wild-type (wt) Cre. (**A**) Schematics of three Cre proteins with a His tag at the C-terminus; (**B**) SDS-PAGE assay for purity of RVG-Cre, Tat-Cre, and wt Cre proteins; (**C**) Schematics of linearized pDFR plasmid after RVG-Cre-, Tat-Cre-, or wt Cre-mediated recombination. pDFR plasmid contains two loxP sites (blue arrowheads). After Cre-mediated recombination, pDFR is cleaved into a 5692 bp linearized fragment (pDFR-I) and a 2652 bp plasmid (pDFR-C); (**D**) Agarose gel electrophoresis of linearized pDFR recombined by RVG-Cre, Tat-Cre, or wt Cre. The two bands of pDFR-C represent nicked and supercoiled conformations of a circular plasmid, respectively; (**E**) Cell penetration of FITC-labeled RVG-Cre, Tat-Cre, or wt Cre in Neuro2a versus HeLa cells where 5 × 10^5^ Neuro2a cells and 5 × 10^5^ HeLa cells were plated in a 48-well plate, and cultured at 5% CO_2_ overnight, followed by treatment with 1 µM FITC-labeled RVG-Cre, Tat-Cre, or wt Cre protein; Scale bar: 100 µM. (**F**) Percent of FITC positive cells treated with FITC-labeled RVG-Cre, Tat-Cre, or Cre proteins. All data are represented as mean ± S.D., *n* = 3. One way ANOVA was used for statistics. * *p* < 0.01.

To examine the in vitro properties of RVG-Cre, we first constructed an RVG-Cre expression vector, along with control constructs for wild-type (wt) Cre and a previously reported TAT-Cre (Figure 1A,B). We next tested the recombinase activity of these three Cre proteins. They were all able to cut a test BsaI linearized plasmid pDFR, which contains two loxP sites and can be used to test Cre recombinase, into a 5692 bp linearized fragment pDFR-I and a 2652 bp circular plasmid pDFR-C (Figure 1C,D). This indicates that fusion of RVG does not change the recombinase activity of the Cre enzyme.

### 2.2. RVG-Cre Cell Line Preference

To test whether RVG-Cre has differential preferences for cultured cell lines, we added labeled RVG-Cre, Tat-Cre, or wt Cre to cultured Neuro2a and HeLa cells. After a 4 h incubation followed by heparin treatment to wash out non-specific attachment, FITC-RVG-Cre clearly showed a strong preference for Neuro2a cells over HeLa cells (Figure 1E,F). FITC-Tat-Cre exhibited no selectivity among the two cell lines, while FITC-Cre showed a similar low affinity for both cell lines. These results suggest that RVG-Cre indeed has greater specificity for Neuro2a cells. The results do not distinguish preferential cell attachment from cell uptake. To demonstrate preferential cell uptake, a Cre reporter cassette should be used, but one is not available.

### 2.3. RVG-Cre Functions in mTmG Mice

**Figure 2:**
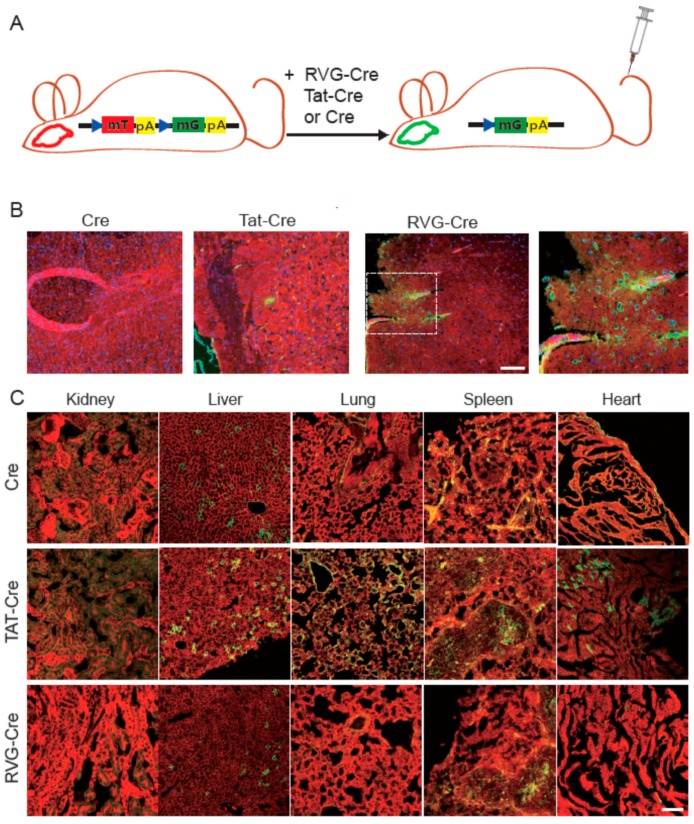
RVG-Cre mediates efficient recombination in the brain after tail vein injection. (**A**) Schematic showing the fluorescence switching principle of the mTmG Cre reporter mouse line. RVG-Cre, Tat-Cre, Cre were administered systemically through tail vein injection on three consecutive days. GFP is shown in green and tdTomato is shown in red; (**B**) Brain sections of wt Cre, Tat-Cre and RVG-Cre injected into mTmG mice. Enlarged view of the dashed box is shown; (**C**) Fluorescence microscopy of other tissues from wt Cre, Tat-Cre and RVG-Cre injected mTmG mice (*n* = 3). Scale bar is 200 µM.

To test whether RVG-Cre functions in vivo, mTmG Cre reporter mice [13] were injected with RVG-Cre, Tat-Cre or wt Cre. Before Cre-mediated recombination, all cells express a transmembrane form of tdTomato fluorescent protein (mT). After successful Cre-mediated recombination, the mT expression switches to the transmembrane form of GFP (mG) expression (Figure 2A). Compared with TAT-Cre and wt Cre, RVG-Cre injection showed more efficient recombination in the brain of mTmG mice (Figure 2B), indicating that RVG-Cre could pass through the BBB and mediate genome editing in brain cerebral cortex cells. While the in vitro results did not distinguish preferential cell attachment from the cell uptake of RVG-Cre, the in vivo results are consistent with preferential cell uptake.

### 2.4. Tissue Specificity of RVG-Cre In Vivo

To examine the tissue specificity of RVG-Cre targeting, we harvested organs of the injected mice. RVG-Cre exhibited little penetrating ability in the heart and lungs. In the liver, spleen and kidney, some recombination events could be detected, but to a lower extent than control mice injected with Tat-Cre or wt Cre. These results suggest that RVG-Cre is the most specific Cre for brain targeting among those tested, although targeting other tissues cannot be completely avoided. (Figure 2C).

### 2.5. Confirmation of Cre Targeting and Activity in Rosa26^lacZ^ Mice

To further confirm the in vivo results from mTmG mice, we performed similar RVG-Cre injection experiments with Rosa26^lacZ^ mice, which are another Cre reporter mouse line where lacZ is only expressed after Cre-mediated recombination [14]. Whole-mount X-gal staining and subsequent sectioning showed that RVG-Cre chiefly targeted cerebral cortex cells, effecting Cre/loxP recombination and lacZ expression. In contrast, Tat-Cre targeting to the brain was much weaker, consistent with the results obtained from the injection of mT/mG mice. (Figure S1).

## 3. Discussion

We have demonstrated in this study that recombinant RVG-Cre protein can pass through the BBB and achieve gene recombination in the brain of mTmG and Rosa26^lacZ^ mice. Because the BBB is notoriously difficult to traverse, genome editing has not been readily performed in the brain. Compared with other previously reported CPP-Cre proteins, our RVG-Cre protein exhibited a much clearer brain specificity for genome editing [15,16,17]. Now, new genome-editing nucleases ZFN, TALEN and CRISPR/Cas9 are becoming powerful genetic tools for biological and medical applications. They may be fused with tissue-specific CPPs such as RVG for more specific in vivo gene therapy as a safer approach than viral or non-viral DNA methods [18,19].

## 4. Materials and Methods

### 4.1. Cloning, Overexpression and Purification of WT Cre, TAT-Cre and RVG-Cre

The bacterial expression plasmid pET-28.2 was purchased from Addgene. It encodes the TAT-Cre with a C-terminus His tag and was used as a control. To create pET-RVG-Cre plasmid, synthesized oligonucleotides encoding the RVG29 peptide were subcloned in frame of Cre in pET-28.2. We also removed the TAT sequence in pET-28.2, and the resulting plasmid pET-wtCre was used as a wild-type Cre control. *E. coli* strain BL21 (DE3) was used for protein expression for all three plasmids. Protein expression was induced with 0.5 mM IPTG for 16 h at 16 °C. Wt Cre, TAT-Cre and RVG-Cre were purified using Ni-NTA resin (Qiagen, Hilden, Germany) and cation-exchange chromatography (HiTrap SP HP) (GE Healthcare, Uppsala, Sweden) according to the manufacturer’s manual. The protein purity was examined by SDS-PAGE. And protein concentration was determined with Bradford assay and adjusted to ~1 µg/µL. Proteins were stored at −80 °C.

### 4.2. Enzyme Activity Assay of Three Cre Fusion Proteins In Vitro

To test Cre functionality in vitro, we constructed a reporter plasmid pDFR based on the pUC19 backbone with two loxP sites for Cre-mediated recombination in vitro. BsaI linearized pDFR plasmid 250 ng was recombined with wt Cre, TAT-Cre or RVG-Cre. We also compared the activities of different concentrations of each Cre protein. The reactions were incubated at 37 °C for 30 min and then heated at 70 °C for 15 min to inactivate the enzyme. Successful recombination of the linearized pDFR plasmid generated a linearized plasmid of 5692 bp and a cyclic plasmid of 2652 bp. Cre enzyme from NEB was used as a positive control.

### 4.3. Transduction of FITC-Labeled Recombinant Proteins into Cells

Neuro2a and HeLa cell lines were used to examine in vitro features of RVG-Cre compared with TAT-Cre and wt Cre. We labeled these Cre proteins with FITC at a final concentration of 0.5 µg/µL. Cells were seeded into 48-well plates at 5000 per well, and kept at 37 °C in a humidified atmosphere containing 5% CO_2_. Then we incubated cells with FITC-labeled Cre proteins for 4 h. After incubation for 4 h, cells were treated with PBS containing 25 µg/µL heparin three times [20]. Hoechst 33342 staining was performed after protein incubation to visualize nuclei. Cells were imaged using a fluorescent microscope (Nikon, Tokyo, Japan).

### 4.4. In Vivo Test of Cre Proteins with mTmG and Rosa26^lacZ^ Mouse Lines

All mouse experiments in this study were approved by the institutional animal care and use committee at China Agricultural University. Approval No. SKLAB-2016-01-07. Approval date (07/01/2016). RVG-Cre, TAT-Cre and Cre were injected via tail vein into mTmG and *Rosa26^lacZ^* mice at 25 µg/g body weight for three consecutive days in 1 mL of PBS, respectively [14]. Each protein was tested with three successfully injected experimental animals in each mouse line (*n* = 3).

## Supplementary Materials

Supplementary materials can be found at www.mdpi.com/1422-0067/17/12/2104/s1.

## Abbreviations

CPP	Cell penetrating peptide
BBB	blood-brain barrier
RVG	rabies virus glycoprotein
wt	wild type
mT	a membrane form tdTomato fluorescent protein
mG	a membrane form GFP fluorescent protein

## References

[1] Milletti F. (2012). Cell-penetrating peptides: Classes, origin, and current landscape. Drug Discov. Today.

[2] Gao Y., Wang Z.Y., Zhang J., Zhang Y., Huo H., Wang T., Jiang T., Wang S. (2014). RVG-peptide-linked trimethylated chitosan for delivery of siRNA to the brain. Biomacromolecules.

[3] Lindsay M.A. (2002). Peptide-mediated cell delivery: Application in protein target validation. Curr. Opin. Pharmacol..

[4] Schwarze S.R., Ho A., Vocero-Akbani A., Dowdy S.F. (1999). In vivo protein transduction: Delivery of a biologically active protein into the mouse. Science.

[5] Kondo E., Saito K., Tashiro Y., Kamide K., Uno S., Furuya T., Mashita M., Nakajima K., Tsumuraya T., Kobayashi N. (2012). Tumour lineage-homing cell-penetrating peptides as anticancer molecular delivery systems. Nat. Commun..

[6] Yu P., Liu B., Kodadek T. (2005). A high-throughput assay for assessing the cell permeability of combinatorial libraries. Nat. Biotechnol..

[7] Kumar P., Wu H., McBride J.L., Jung K.E., Kim M.H., Davidson B.L., Lee S.K., Shankar P., Manjunath N. (2007). Transvascular delivery of small interfering RNA to the central nervous system. Nature.

[8] Tuffereau C., Schmidt K., Langevin C., Lafay F., Dechant G., Koltzenburg M. (2007). The rabies virus glycoprotein receptor p75NTR is not essential for rabies virus infection. J. Virol..

[9] Xiang L., Zhou R., Fu A., Xu X., Huang Y., Hu C. (2011). Targeted delivery of large fusion protein into hippocampal neurons by systemic administration. J. Drug Target..

[10] Liu Y., Huang R., Han L., Ke W., Shao K., Ye L., Lou J., Jiang C. (2009). Brain-targeting gene delivery and cellular internalization mechanisms for modified rabies virus glycoprotein RVG29 nanoparticles. Biomaterials.

[11] Hamilton D.L., Abremski K. (1984). Site-specific recombination by the bacteriophage P1 lox-Cre system. Cre-mediated synapsis of two lox sites. J. Mol. Biol..

[12] Nagy A. (2000). Cre recombinase: The universal reagent for genome tailoring. Genesis.

[13] Muzumdar M.D., Tasic B., Miyamichi K., Li L., Luo L. (2007). A global double-fluorescent Cre reporter mouse. Genesis.

[14] Soriano P. (1999). Generalized lacZ expression with the ROSA26 Cre reporter strain. Nat. Genet..

[15] Jo D., Nashabi A., Doxsee C., Lin Q., Unutmaz D., Chen J., Ruley H.E. (2001). Epigenetic regulation of gene structure and function with a cell-permeable Cre recombinase. Nat. Biotechnol..

[16] Chien W.M., Liu Y., Chin M.T. (2014). Genomic DNA recombination with cell-penetrating peptide-tagged Cre protein in mouse skeletal and cardiac muscle. Genesis.

[17] Cronican J.J., Thompson D.B., Beier K.T., McNaughton B.R., Cepko C.L., Liu D.R. (2010). Potent delivery of functional proteins into Mammalian cells in vitro and in vivo using a supercharged protein. ACS Chem. Biol..

[18] Gitton Y., Tibaldi L., Dupont E., Levi G., Joliot A. (2009). Efficient CPP-mediated Cre protein delivery to developing and adult CNS tissues. BMC Biotechnol..

[19] Zuris J.A., Thompson D.B., Shu Y., Guilinger J.P., Bessen J.L., Hu J.H., Maeder M.L., Joung J.K., Chen Z.Y., Liu D.R. (2015). Cationic lipid-mediated delivery of proteins enables efficient protein-based genome editing in vitro and in vivo. Nat. Biotechnol..

[20] Richard J.P., Melikov K., Brooks H., Prevot P., Lebleu B., Chernomordik L.V. (2005). Cellular uptake of unconjugated TAT peptide involves clathrin-dependent endocytosis and heparan sulfate receptors. J. Biol. Chem..

