# Supplementary material for: Cre Fused with RVG Peptide Mediates Targeted Genome Editing in Mouse Brain Cells In Vivo
Source: Int J Mol Sci. 2016 Dec 14;17(12):2104. doi: 10.3390/ijms17122104 (PMC5187904; doi:10.3390/ijms17122104)
Supplement: Supplementary file 1 [file ijms-17-02104-s001.pdf]

Supplementary Materials: Cre Fused with RVG Peptides Mediates Targeted Genome Editing in Mouse Brain Cells In Vivo

Zhiyuan Zou, Zhaolin Sun, Pan Li, Tao Feng and Sen Wu

Table S1. List of oligos for pET-RVG-Cre and pET-wtCre construction.

Oligo ID	Oligo Sequence
RVG-Cre-F	5′-GAGATATAccatgggcTACACCATCTGGATGCCGGAAAACCCGCGTCCGGGTACCCCG TGCGACATCTTCACCAACTCTCGTGGTAAACGTGCTTCTAACGGTGGTGGTGGTggcca-3′
RVG-Cre-R	5′-ggcTACACCATCTGGATGCCGGAAAACCCGCGTCCGGGTACCCCGTGCGACATCT TCACCAACTCTCGTGGTAAACGTGCTTCTAACGGTGGTGGTGGTggccata-3′
Cre-F	5′-catgggcggcca-3′
Cre-R	5′-tatggccgcc-3′

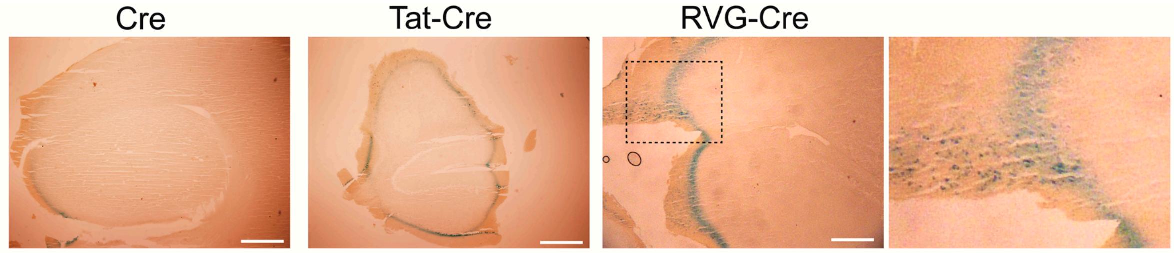

Figure S1. Sections of whole-mount brain staining from Rosa26^{lacZ} mice injected with RVG-Cre, TAT-Cre and Cre proteins, respectively (n = 3). RVG-Cre mainly targeted to the cerebral cortex. Scale bar is 200 μM.

Full sequences of RVG-Cre Cassette:

5′-atgggcTACACCATCTGGATGCCGGAAAACCCGCGTCCGGGTACCCCGTGCGACATCTTC
ACCAACTCTCGTGGTAAACGTGCTTCTAACGGTGGTGGTGGTggccatatggctagcatgactggtggacag
caaatgggtcgggatccgaattccatgtccaatttactgaccgtacaccaaaatttgcctgcattaccggtcgatgcaacgagtgatgaggttcgc
aagaacctgatggacatgttcagggatcgccaggcgttttctgagcatacctggaaaatgcttctgtccgtttgccggtcgtgggcggcatggtgc
aagttgaataaccggaaatggtttcccgcagaacctgaagatgttcgcgattatcttctatatcttcaggcgcgcggtctggcagtaaaaactatcc
agcaacatttgggccagctaaacatgcttcatcgtcggtccgggctgccacgaccaagtgacagcaatgctgtttcactggttatgcggcggatcc
gaaaagaaaacgttgatgccggtgaacgtgcaaaacaggctctagcgttcgaacgcactgatttcgaccaggttcgttcactcatggaaaatagc
gatcgctgccaggatatacgtaatctggcatttctggggattgcttataacaccctgttacgtatagccgaaattgccaggatcagggttaaagata
tctcacgtactgacggtgggagaatgttaatccatattggcagaacgaaaacgctggttagcaccgcaggtgtagagaaggcacttagcctggg
ggtaactaaactggtcgagcgatggatttccgtctctggtgtagctgatgatccgaataactacctgttttgccgggtcagaaaaaatggtgttgcc
gcgccatctgccaccagccagctatcaactcgcgccctggaagggatttttgaagcaactcatcgattgatttacggcgctaaggatgactctggt
cagagatacctggcctggtctggacacagtgcccgtgtcggagccgcgcgagatatggcccgcgctggagtttcaataccggagatcatgcaa
gctggtggctggaccaatgtaaatattgtcatgaactatatccgtaacctggatagtgaaacaggggcaatggtgcgcctgctggaagatggcg
atgcggccgcactcgagcaccaccaccaccaccactga-3′

Full Sequences of TAT-Cre Cassette:

5′-atgggcaggaagaagcggagacagcgacgaagaggccatatggctagcatgactggtggacagcaaatgggtcgggatccgaattc
catgtccaatttactgaccgtacaccaaaatttgcctgcattaccggtcgatgcaacgagtgatgaggttcgcaagaacctgatggacatgttcag
ggatcgccaggcgttttctgagcatacctggaaaatgcttctgtccgtttgccggtcgtgggcggcatggtgcaagttgaataaccggaaatggtt
tcccgcagaacctgaagatgttcgcgattatcttctatatcttcaggcgcgcggtctggcagtaaaaactatccagcaacatttgggccagctaaac
atgcttcatcgtcggtccgggctgccacgaccaagtgacagcaatgctgtttcactggttatgcggcggatccgaaaagaaaacgttgatgccgg
tgaacgtgcaaaacaggctctagcgttcgaacgcactgatttcgaccaggttcgttcactcatggaaaatagcgatcgctgccaggatatacgtaa
tctggcatttctggggattgcttataacaccctgttacgtatagccgaaattgccaggatcagggttaaagatatctcacgtactgacggtgggaga
atgttaatccatattggcagaacgaaaacgctggttagcaccgcaggtgtagagaaggcacttagcctgggggtaactaaactggtcgagcgat

ggatttccgtctctggtgtagctgatgatccgaataactacctgttttgccgggtcagaaaaaatggtgttgccgcgccatctgccaccagccagct
atcaactcgcgccctggaagggatttttgaagcaactcatcgattgatttacggcgctaaggatgactctggtcagagatacctggcctggtctgg
acacagtgcccgtgtcggagccgcgcgagatatggcccgcgctggagtttcaataccggagatcatgcaagctggtggctggaccaatgtaaat
attgtcatgaactatatccgtaacctggatagtgaaacaggggcaatggtgcgcctgctggaagatggcgatgcggccgcactcgagcaccacc
accaccaccactga-3′

Full Sequences of Cre Cassette:

5′-atgggcggccatatggctagcatgactggtggacagcaaatgggtcgggatccgaattccatgtccaatttactgaccgtacaccaaaatttg
cctgcattaccggtcgatgcaacgagtgatgaggttcgcaagaacctgatggacatgttcagggatcgccaggcgttttctgagcatacctggaa
aatgcttctgtccgtttgccggtcgtgggcggcatggtgcaagttgaataaccggaaatggtttcccgcagaacctgaagatgttcgcgattatctt
ctatatcttcaggcgcgcggtctggcagtaaaaactatccagcaacatttgggccagctaaacatgcttcatcgtcggtccgggctgccacgacca
agtgacagcaatgctgtttcactggttatgcggcggatccgaaaagaaaacgttgatgccggtgaacgtgcaaaacaggctctagcgttcgaac
gcactgatttcgaccaggttcgttcactcatggaaaatagcgatcgctgccaggatatacgtaatctggcatttctggggattgcttataacaccctg
ttacgtatagccgaaattgccaggatcagggttaaagatatctcacgtactgacggtgggagaatgttaatccatattggcagaacgaaaacgct
ggttagcaccgcaggtgtagagaaggcacttagcctgggggtaactaaactggtcgagcgatggatttccgtctctggtgtagctgatgatccg
aataactacctgttttgccgggtcagaaaaaatggtgttgccgcgccatctgccaccagccagctatcaactcgcgccctggaagggatttttgaa
gcaactcatcgattgatttacggcgctaaggatgactctggtcagagatacctggcctggtctggacacagtgcccgtgtcggagccgcgcgag
atatggcccgcgctggagtttcaataccggagatcatgcaagctggtggctggaccaatgtaaatattgtcatgaactatatccgtaacctggata
gtgaaacaggggcaatggtgcgcctgctggaagatggcgatgcggccgcactcgagcaccaccaccaccaccactga-3′
